# 

**Published:** 1914-03-07

**Authors:** 


### ASSISTANT MENTAL MEDICAL OFFICERS.

Dr. F. J. Stuart, Hon. Secretary of the Association of Assistant Medical Officers of Mental Hospitals, England and Wales, asks us to state that the first annual general meeting of the Association will be held at the Westminster Palace Hotel, London, on Saturday, March 14, at 3.15 p.m.

### Gifts and Bequests.

The Secretary of Ancoats Hospital, Manchester, received recently a bank-note for £100 with a note worded: "Please insert this amount in your report as from B. A."

Louisa Mary Lady Knightley of Fawsley, of Northants, has left £1,000 to Northampton Hospital for the endowment of a "Rainald Knightley" Bed in memory of her husband, Lord Knightley.

RATES OF SUBSCRIPTION: Twelve months, 6/6; Six months, 4/-; Three months, 2/-, post free to any address in the United Kingdom. Abroad, Twelve months, 8/8; Six months, 5/-; Three months, 2/6, post free.—Address The Subscription Dept., "THE HOSPITAL," The Hospital Building, 28/29 Southampton Street, Strand, London, W.C.

